# On the nucleus composition during isothermal alloy solidification

**DOI:** 10.1038/s41598-018-23123-w

**Published:** 2018-03-28

**Authors:** Xun Kang, Lijun Zhang, Sergey Sobolev

**Affiliations:** 1 State Key Laboratory of Powder Metallurgy, Central South University, Changsha, Hunan 410083 China; 2 Institute of Problems of Chemical Physics, Academy of Sciences of Russia, Chernogolovka, Moscow Region, 14232 Russia

## Abstract

Accurate determination of the nucleus composition during isothermal alloy solidification still represents a great challenge nowadays. In this paper, a kinetic scheme was added to the Hillert-Rettenmayr thermodynamic analysis of the deviation from local equilibrium at migrating phase interfaces. A so-called interface permeability was introduced to account for the unambiguous determination of the energy dissipation of the solute rearrangement at the liquid-solid interface and the driving force for interface movement, from which the nucleus composition can be then evaluated. After benchmark test, a pragmatic nucleation model for solidification was also proposed, and applied in three-dimensional phase-field simulations of nucleation and subsequent dendritic growth during isothermal solidification process in one hypothetic Al-Si alloy. Moreover, the influence of the interface permeability on nucleation rate was fully discussed by exploring its effect on the initial nucleus components and the corresponding nucleation driving force.

## Introduction

When alloy melt cools down continuously or cools rapidly to a certain temperature with certain undercooling, nucleus of primary solid phase may start to form in the melt, and thus trigger the evolution of microstructure during the solidification process. Nucleation is the prior stage during the solidification process, and draws numerous attentions in the field of materials^1,2^. In order to gain a comprehensive and quantitative view of nucleation process in alloys, accurate determination of the composition for nucleus, especially for the first nucleus, during solidification is prerequisite, which still remains a challenge in experimental and theoretical investigations nowadays^3^.

**Figure 1:**
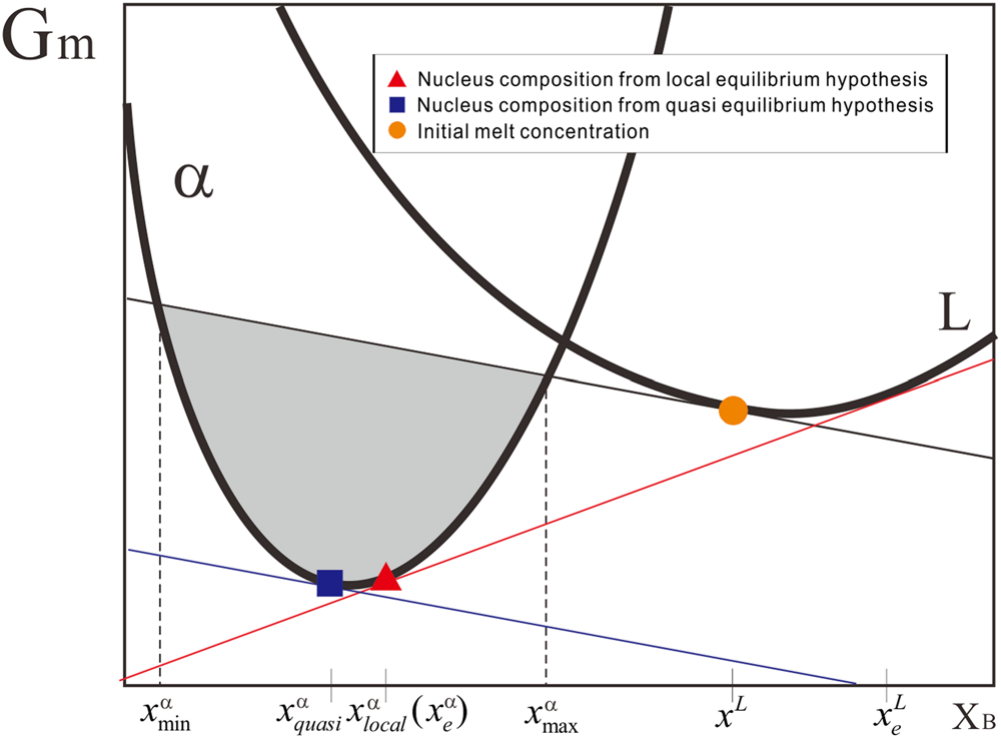
Schematic diagram of determination of nucleus composition during isothermal solidification based on different thermodynamic hypotheses.

Baker and Cahn^4^ firstly explored the thermodynamically possible composition range for nucleus, as demonstrated in Fig. 1.

Assuming in a fictitious binary A-B alloy during isothermal solidification, ${x}_{local}^{\alpha }$ and ${x}_{e}^{L}$ are the equilibrium compositions while ${x}^{L}$ is the initial composition of alloy melt. Baker and Cahn^4^ concluded that it would be thermodynamically possible to nucleate α with any composition between ${x}_{\min }^{\alpha }$ and ${x}_{\max }^{\alpha }$ from the melt. In order to further determine the specific composition for the nucleus, two hypotheses are generally used. One is the well-known local equilibrium hypothesis (i.e., the common tangent construction)^5^, as schematically described in Fig. 1. Under the local equilibrium hypothesis, the chemical potentials of the solid and liquid phases should be equal, and thus the composition of α nucleus from melt is exactly the equilibrium composition ${x}_{local}^{\alpha }$. While the other is the so-called quasi equilibrium hypothesis (i.e., the parallel tangent condition)^6^, as also demonstrated in Fig. 1. Under the quasi equilibrium hypothesis, the diffusion potentials of the solid and liquid phases are required to be equal, and thus the composition of α nucleus from melt should be ${x}_{quasi}^{\alpha }$. Though the above two hypotheses are widely used in the field of materials, the nucleation process is simply treated to be either equilibrium or near equilibrium state. While in practice, the nucleation process should be non-equilibrium and even strongly non-equilibrium in some cases.

On purpose of precisely determining the nucleus composition, one approach is in great need for describing the non-equilibrium process by suspending the above-mentioned thermodynamic hypotheses, which is the major target of this work. Recently, Hillert and Rettenmayr^7^ gave a thermodynamic view of deviation from local equilibrium at migrating phase interfaces. Their constructions recapitulated that a part of the driving force is consumed *via* the exchange of solute atoms between two phases over the interface, resulting in a deviation from the local equilibrium. With the Hillert-Rettenmayr analysis, one may analyze the driving force for the non-equilibrium nucleation process, which is the necessity for the later quantitative description of microstructure evolution during solidification process. However, before the unambiguous determination of driving forces for non-equilibrium nucleation process, the accurate nucleus composition should be given. However, the thermodynamic alone cannot predict the actual composition of nucleus.

Consequently, a kinetic scheme will be added on the Hillert-Rettenmayr thermodynamic analysis in this paper, from which the nucleus composition for alloys during isothermal solidification can be unambiguously determined. Furthermore, based on the thermodynamic and kinetic analysis, a new but pragmatic nucleation model for isothermal solidification is thus to be proposed, and further applied to simulate the nucleation and dendritic growth process in a binary Al-Si alloy using the phase-field model with finite interface dissipation^8^.

## Results and Discussion

### Hillert-Rettenmayr thermodynamic analysis

During the formation of precipitation solid *α* from a supersaturated liquid *L*, a local decrease in Gibbs energy is yielding

1 $${\rm{\Delta }}{G}_{m}^{{\rm{t}}{\rm{o}}{\rm{t}}{\rm{a}}{\rm{l}}}=(1-{x}^{\alpha })({\mu }_{A}^{L}-{\mu }_{A}^{\alpha })+{x}^{\alpha }({\mu }_{B}^{L}-{\mu }_{B}^{\alpha })=(1-{x}^{L})({\mu }_{A}^{L}-{\mu }_{A}^{\alpha })+{x}^{L}({\mu }_{B}^{L}-{\mu }_{B}^{\alpha })+({x}^{L}-{x}^{\alpha })({\mu }_{A}^{L}-{\mu }_{A}^{\alpha }-{\mu }_{B}^{L}+{\mu }_{B}^{\alpha })$$

where *x*^*α*^ and *x*^*L*^ are the local compositions at the interface of *α* and *L*. ${\mu }_{A}^{L}$ and ${\mu }_{B}^{L}$ are the chemical potentials of species A and B in liquid, while ${\mu }_{A}^{\alpha }$ and ${\mu }_{B}^{\alpha }$ are treated analogously in solid *α*.

**Figure 2:**
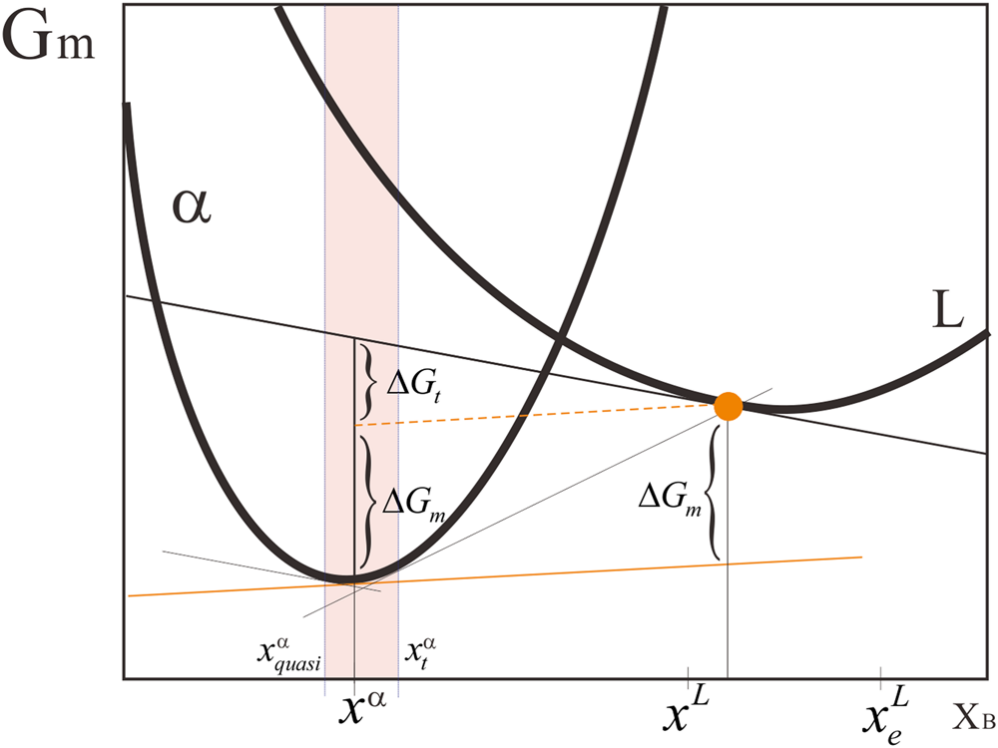
Driving forces for adjustment of A and B atoms between two phases, Δ*G*_*t*_, and, Δ*G*_*m*_, for precipitation of *α* from a supersaturated liquid ${x}^{L}$.

According to Hillert and Rettenmayr^7^, the phase transformation during solidification can be divided into two detached processes. One is the transformation from liquid *L* with composition *x*^*L*^ to solid *α* with composition *x*^*L*^, while the other is the adjustment of the composition from *x*^*L*^ to *x*^*α*^ by exchanging atoms between the two phases *via* diffusion over the interface, as shown in Fig. 2. Thus, one can separate the total driving force into the fraction ${\rm{\Delta }}{G}_{m}$ which drives the phase transformation and the fraction ${\rm{\Delta }}{G}_{t}$ which drives the redistribution of atoms between the phases,

2 $${\rm{\Delta }}{G}_{m}^{total}={\rm{\Delta }}{G}_{t}+{\rm{\Delta }}{G}_{m}$$

3 $${\rm{\Delta }}{G}_{m}=({\mu }_{A}^{L}-{\mu }_{A}^{\alpha })-{x}^{L}\cdot {\rm{\Delta }}\tilde{\mu }$$

4 $${\rm{\Delta }}{G}_{t}={\rm{\Delta }}x\cdot {\rm{\Delta }}\tilde{\mu }$$

Here, ${\rm{\Delta }}\tilde{\mu }=({\mu }_{A}^{L}-{\mu }_{A}^{\alpha }-{\mu }_{B}^{L}+{\mu }_{B}^{\alpha })$ indicates the diffusion potential difference of two phases. ${\rm{\Delta }}x={x}^{L}-{x}^{\alpha }$ means the difference in concentrations between the two phases.

With the Hillert-Rettenmayr thermodynamic analysis, *x*^*L*^ is the initial alloy composition, and is known a priori. Accordingly, the chemical potentials ${\mu }_{A}^{L}$ and ${\mu }_{B}^{L}$ can also be derived. While in Eqs (3) and (4), *x*^*α*^, ${\mu }_{A}^{\alpha }$ and ${\mu }_{B}^{\alpha }$ are unknown, but ${\mu }_{A}^{\alpha }$ and ${\mu }_{B}^{\alpha }$ depend on the nucleus composition *x*^*α*^. Thus, one can clearly separate ${\rm{\Delta }}{G}_{m}^{{\rm{total}}}$, Δ*G*_*m*_ and Δ*G*_*t*_ for specific alloy composition if the nucleus composition is known. But Eqs (3) and (4) are not enough to evaluate *x*^*α*^. Instead, only a corrected nucleus concentration range can be gained based on the constraint that both Δ*G*_*m*_ and Δ*G*_*t*_ cannot be less than zero. Thus, one needs more profound analysis to fix the freedom, from the perspective of kinetics.

### Kinetic analysis

**Figure 3:**
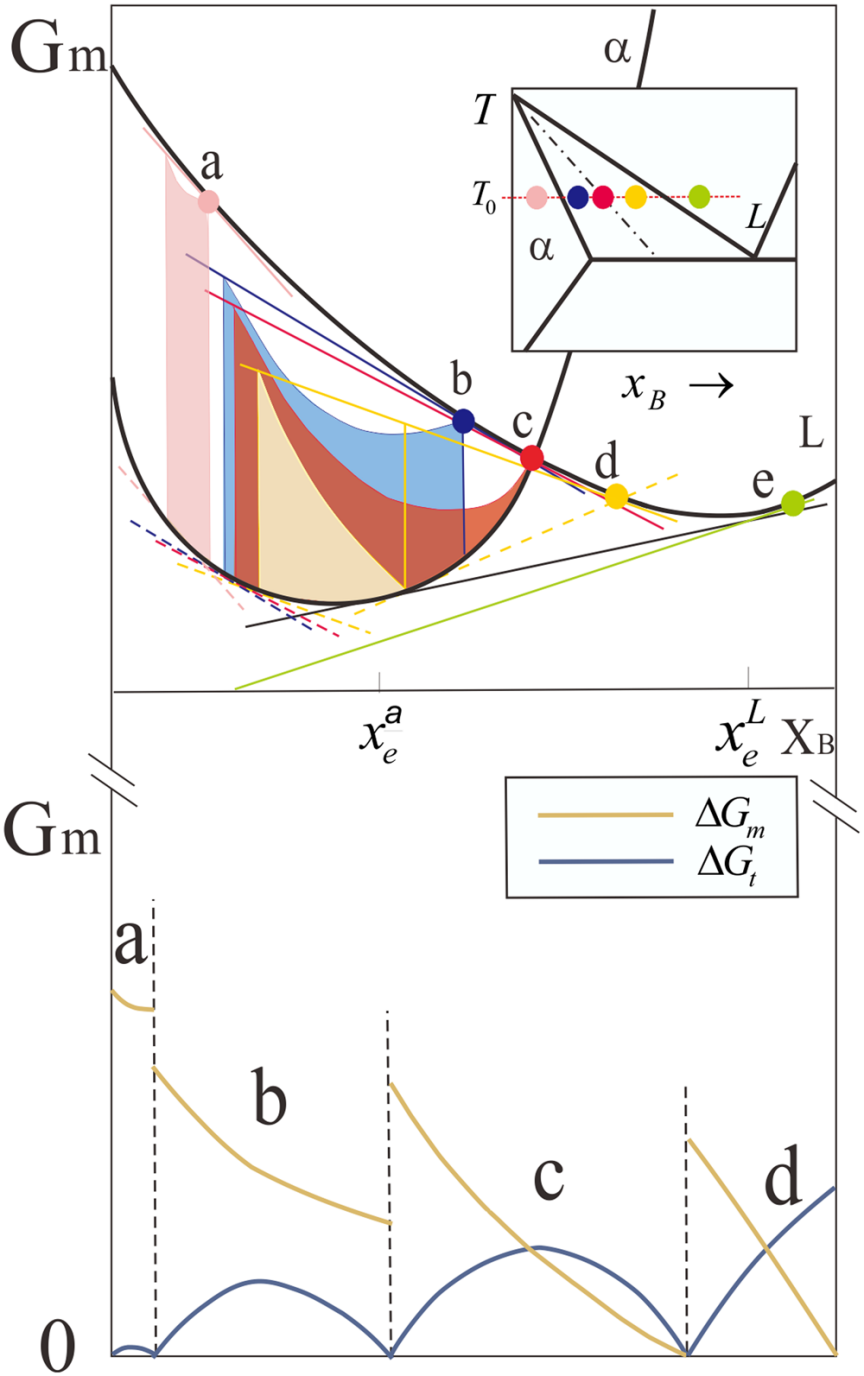
Limits of nucleus composition and distribution of driving force corresponding to different initial alloy melts.

Taking a fictitious A-B binary system as an example, five different alloy compositions, i.e., *a*, *b*, *c*, *d* and *e*, at one constant temperature are chosen for demonstration, as presented in Fig. 3. Point *a* lies on left side of solidus line, point *b* lies between solidus line and T_0_ line, point *c* lies on T_0_ line, point *d* lies between T_0_ line and liquidus line, while point *e* lies on the right side of liquidus line. The possible nucleus concentration ranges corresponding to different initial melt concentrations based on the Hillert-Rettenmayr thermodynamic analysis are obtained and illustrated schematically with the molar Gibbs energy diagrams in Fig. 3. It is well known that the vertical distances represent the driving forces for the precipitation of nucleus with various compositions from the melt, which are composed of Δ*G*_*m*_ and Δ*G*_*t*_ for each supersaturated melt concentration. As displayed in Fig. 3, the colored part characterizes Δ*G*_*m*_ and the blank part is Δ*G*_*t*_. For clarity, the distribution of driving force information is sorted out and shown underneath the graph. Next this diagram will be analyzed and elaborated from the kinetic view.

For this purpose, a physical kinetic parameter named the interface permeability for redistribution flux or the inverse interface resistivity, *P*, originally defined in the phase-field model with finite interface dissipation^8,9^, is directly used here. According to refs^8,9^, *P* has the units of an inverse action density (cm^3^/(Js)), which is used to characterize the ability of atoms to overcome resistance during migration inside interface. When interface permeability *P* equals to zero, it indicates that the rearrangement resistance is huge enough to nail the solute atoms. While on the contrary, if the interface permeability *P* approaches to infinity (or larger enough), the solute rearrangement will be almost free from resistance, and the diffusion potential difference will approach zero instantaneously. Then the impact of atomic mobility on dissipation of the solute rearrangement and driving force for interface migration can be explained expediently. The discussion will be divided into three cases on the basis of the solidification conditions:

#### Case 1 (point d)

The interface migration from liquid to solid is torpid, which implies that a liquid atom has enough time and enough trials to move to find its place in the new crystal structure of solid phase. While the permeability approaches to be infinite, the driving force consumed for the motion of the solute atoms is inexistent but dissipated in the phase transition completely. That is to say, Δ*G*_*t*_ = 0 while Δ*G*_*m*_ is maximum here. With the permeability decreasing, the atomic mobility resistance will increase, the driving force consumed in solute rearrangement will increase and the available driving force for phase transition will be reduced. Furthermore, when the resistance is large enough, the driving force will be exhausted entirely by the rearrangement and motion of solute atoms over the interface, i.e., that Δ*G*_*t*_ maximum and ${\rm{\Delta }}{G}_{m}=0$.

#### Case 2 (point a)

The interface will be pushed quickly toward liquid phase. There is a certain chance that some solute atoms will be pinned before migration hence the atom mobility is highly sensitive to the resistance in this situation. Therefore, Δ*G*_*t*_ shows a drastically declination compared to the slow solidification since it is positively associated with the product of resistance and the number of moving atoms. If *P* is extremely large, the composition of the precipitate phase will be located at somewhere the diffusion potentials of the two phases are consistent. Furthermore, with the decrease of *P*, the resistance will increase and the quantity of moving solute atoms will decrease, thus the dissipation Δ*G*_*t*_ will increase from zero (i.e., that the resistance is non-existent) to a certain peak and then decreases to zero (i.e., that the solute atoms are all frozen). Besides, the loss of Δ*G*_*m*_ will increase with the deepening of the atomic pinning until a minimum Δ*G*_*m*_ reaches at the point Δ*G*_*t*_ = 0, as illustrated in Fig. 3. It is possible for the clusters to inherit the parental component here.

#### Case 3 (points b and c)

The situation can be treated analogously as Case 2, but the solidification process is slower, Δ*G*_*m*_ is smaller while Δ*G*_*t*_ is larger. Furthermore, it is worth noting that while the melt concentration locates on T_0_ line, if the permeability reduces to zero, both Δ*G*_*m*_ and Δ*G*_*t*_ are equal to zero, which suggests that the system maintains a delicate balance in this situation, where a slight disturbance will cause its rupture.

Next a quantitative determination of the energy dissipation and the driving force for nucleation will be provided.

It is universally acknowledged that the difference of diffusion potential between solid and liquid phases will drive the rearrangement and motion of solute atoms over the interface. The diffusion flux for solute B that crosses the atomistic physical interface can be expressed as:

5 $${J}_{B}=\frac{{M}_{{\rm{inter}}}}{{V}_{m}}\nabla \tilde{\mu }$$

${M}_{{\rm{inter}}}$ is the atomic mobility over the interface. Based on ref.^8^, one can have ${M}_{{\rm{inter}}}=a\eta P/8$, where *a* is the size of the physical solid-liquid interface, and can simply be assumed to be in the same scale of the unit cell of the solidified solid phase, $\eta$ is the thickness of the interface. *V*_*m*_ is the molar volume and $\nabla \tilde{\mu }$ is the gradient in diffusion potential over the interface, which can be estimated as ${\rm{\Delta }}\tilde{\mu }/\eta$ for the interface with a thickness of $\eta$. Alternatively, the diffusion flux for solute B along the moving interface can also be defined as

6 $${J}_{B}=v\cdot {\rm{\Delta }}x$$

7 $$v=M\cdot {\rm{\Delta }}{G}_{m}$$

where *v* is the average velocity of moving interface, Δ*x* is the composition difference of solute in solid and liquid phases over the interface, and *M* is the interface mobility for migrating interface.

Integrating Eqs (4–7), Δ*G*_*t*_ is related to Δ*G*_*m*_ *via*,

8 $${\rm{\Delta }}{G}_{t}=\frac{aP{({\rm{\Delta }}\tilde{\mu })}^{2}}{8{V}_{m}M{\rm{\Delta }}{G}_{m}}$$

Now with the third equation (i.e., Eq. (8)) besides Eqs (3) and (4), one can solve all the unknown quantities in Eqs (3) and (4).

Insert Eqs (3) and (4) into Eq. (8), and the expression for the nucleus composition *x*^*α*^ is emerged:

9 $${x}^{\alpha }={x}^{L}-\frac{aP{\rm{\Delta }}\tilde{\mu }}{8{V}_{m}M[({\mu }_{A}^{L}-{\mu }_{A}^{\alpha })-{x}^{L}\cdot {\rm{\Delta }}\tilde{\mu }]}$$

because both chemical and diffusion potentials (i.e., ${\mu }_{A}^{\alpha }$ and ${\rm{\Delta }}\tilde{\mu }$) are functions of *x*^*α*^ and temperature, *x*^*α*^ can be unambiguously determined from Eq. (9).

Based on Eq. (9), one can arrive at the following conclusions:

i) If interface permeability *P* equals to zero, indicating that the solute atoms stop moving, the nucleus will inherit the composition of the parent liquid phase, ${x}^{\alpha }={x}^{L}$. Under this extreme, Δ*G*_*t*_ = 0 from Eq. (4), and Δ*G*_*m*_ reaches a minimum value since the concentration and diffusion potential difference are maximum here. According to Eq. (3), it can be concluded that the value of the minimum Δ*G*_*m*_ depends on the melt concentration, when it locates on the T_0_ line, ${\rm{\Delta }}{G}_{m}^{\min }=0$; if the concentration lies on the left side of the T_0_ line, ${\rm{\Delta }}{G}_{m}^{\min } > 0$, the concentration inheritance can be achieved with phase transition; while on the right of the T_0_ line ${\rm{\Delta }}{G}_{m}^{\min } < 0$, nucleus will not appear due to the absence of the nucleation driving force.
ii) If the interface permeability *P* approaches to infinity (or larger enough), the situation degrades to the quasi-equilibrium hypothesis, ${x}^{\alpha }={x}_{quasi}^{\alpha }$, ${\rm{\Delta }}{G}_{t}=0$ from Eq. (4), the maximum Δ*G*_*m*_ is reached since the second term in Eq. (5) becomes zero.
iii) When the interface permeability *P* decreases from infinity to zero, *x*^*α*^ will move from ${x}_{quasi}^{\alpha }$ towards *x*^*L*^, the trend of Δ*G*_*m*_ is declining, according to Eq. (5). Whether the nucleation can occur depends on whether the Δ*G*_*m*_ is greater than zero.

### Benchmark test

**Figure 4:**
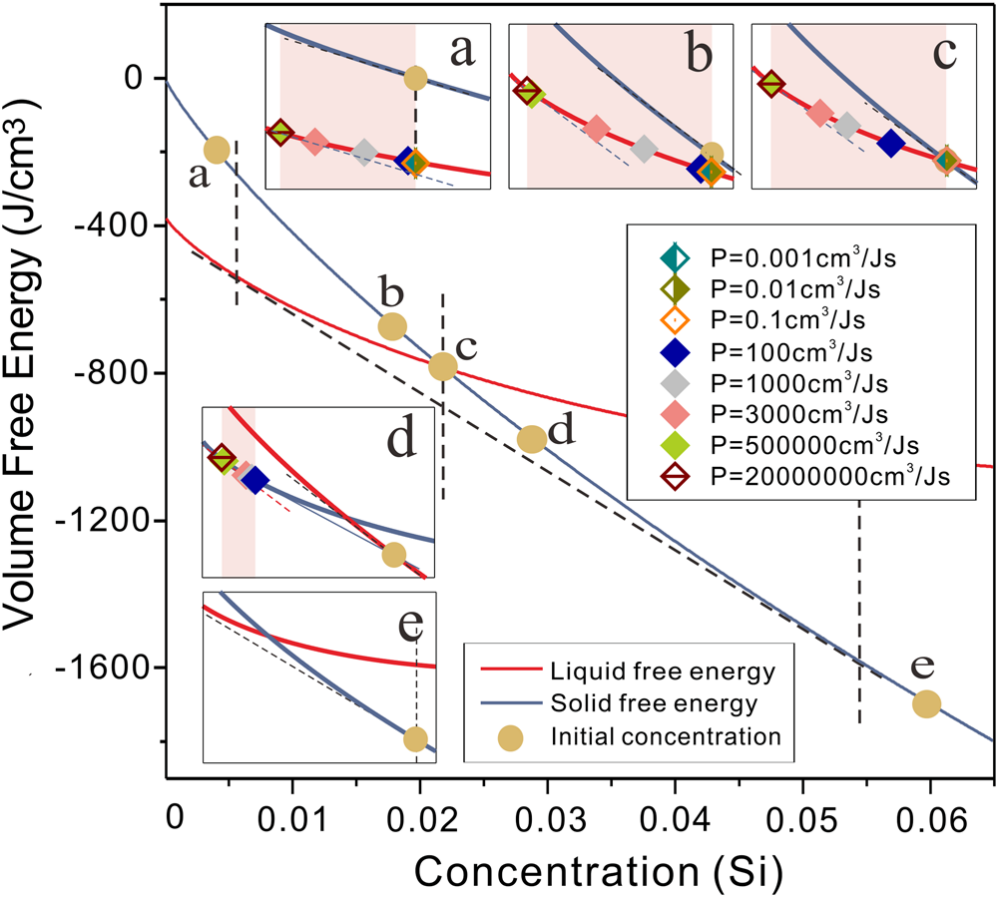
Evaluated nucleus compositions for different Al-Si alloys according to different interfacial permeabilities.

Based on thermodynamic and kinetic analysis demonstrated above, the nucleus composition can be unambiguously determined for a given initial melt composition and interfacial permeability in a specific alloy. In this section, different Al-Si binary alloys were chosen as benchmark test for the above thermodynamic and kinetic analysis. For the sake of simplicity, the linear phase diagram of the binary Al-Si system was utilized, from which the Gibbs energy, chemical/diffusion potentials of both liquid and solid phases can be evaluated^10^. All the relevant thermophysical parameters used in the calculations are listed in Table 1, except for the values of the interface permeability *P*. Here the interface mobility *M* was calculated^11^ to keep the interface movement in the diffusion-control regime. The calculated compositions of nucleus are labeled in Fig. 4. Based on the rigorous analysis of the results in Fig. 4, the following conclusions that the influence of permeability on the new phase composition can be divided into three cases can be drawn,

**Table 1:** List of all the relevant parameters used in the present calculation of nucleus compositions.

Parameters	Symbols	Values
interface mobility	*M*	3.84 × 10^−2^ cm^4^/Js
molar volume	*V*_m_	10.06 cm^3^/mol
melting temperature of solvent Al	*T*_m_	933.6 K
isothermal temperature	*T*	900 K
liquidus slope	*m*_e_	−618.42 K/at.fraction
equilibrium partitioning coefficient	*k*_e_	0.105

#### Case 1: Initial concentration is on the left side of T_0_ line

When *P* varies from infinity to zero, the composition of the first nucleus will move from ${x}_{quasi}^{\alpha }$ to ${x}^{L}$. According to the analysis in the previous section, one know ${\rm{\Delta }}{G}_{m}^{\min } > 0$ here, and hence it is possible for nucleus to form inside the entire composition range from ${x}_{quasi}^{\alpha }$ to ${x}^{L}$.

#### Case 2: Initial concentration is between T_0_ line and liquidus

With the decrease of *P*, the nucleation driving force shows a downward trend. The more complex case lies in that, as the composition moves from ${x}_{quasi}^{\alpha }$ to ${x}^{L}$, the nucleation driving force is reduced from positive to negative, and the nucleus are generated only when the nucleation driving force is greater than zero, leading to the narrowed concentration range of nucleus. Moreover, the calculation results also show that when *P* goes from infinity to a certain threshold greater than zero, a critical component point ${x}_{t}^{\alpha }$ which is in good agreement with the Hillert-Rettenmayr thermodynamic construction will be exposed, and the composition of the nucleus will travel from ${x}_{quasi}^{\alpha }$ to ${x}_{t}^{\alpha }$; while if *P* is less than the minimum limit, the nucleus will not appear.

#### Case 3: Initial concentration is on the right side of liquidus

The nucleation driving force is less with all the *P*s. Thus the formation of nucleus is impossible.

The shaded part in Fig. 4 is the theoretical range given by Hillert *et al*.^7^. Obviously, the calculated results are consistent with the theoretical construction, and the composition of first nucleus can be determined uniquely by *P* within the theoretical range.

### Nucleation model and its application

Based on the previous sections on the evaluation of nucleus composition during isothermal solidification, a pragmatic nucleation model is thus proposed for isothermal solidification in alloys, as demonstrated in the following: During the isothermal solidification, the liquid concentration is not absolutely uniform. The local slight fluctuation in consistency with the Gaussian distribution exists throughout the solidification process. Moreover, once the initial local liquid composition and the interfacial permeability are given, the corresponding nucleus composition and its driving force can be evaluated according to Eqs (3), (4) and (9). Thus, the uneven driving forces for different local liquid compositions will lead to different probabilities of the nucleus in the melt. According to the classical nucleation theory^12^, if the driving force somewhere is large enough to satisfy the condition ${\rm{\Delta }}{G}_{m}\ge 2\sigma /dx$, the nucleus with the radius of *dx* and the concentration of *x*^*α*^ will appear in the melt. After that, the microstructure during isothermal solidification will evolve. Since the concentration fluctuations in the melt always exist during solidification, the nucleus can form continuously as well.

Next, the proposed nucleation model is applied in a real alloy together with the phase-field simulation. Here, one hypoeutectic alloy, Al-2.89 at. % Si, was chosen as the target, and the phase-field model with finite interface dissipation^8^ was utilized in the present work. The evolution equation of the phase field and individual phase concentrations are given^8^:

10 $${\dot{\varphi }}_{\alpha }=\frac{8P\eta M}{8P\eta +M{\pi }^{2}({c}_{\alpha }-{c}_{\beta })}\{\sigma [{{\rm{\Delta }}}^{2}{\varphi }_{\alpha }+\frac{{\pi }^{2}}{{\eta }^{2}}({\varphi }_{\alpha }-\frac{1}{2})]-\frac{\pi }{\eta }\sqrt{{\varphi }_{\alpha }(1-{\varphi }_{\alpha })}{\rm{\Delta }}{G}_{m}\}$$

11 $${\varphi }_{\alpha }{\dot{c}}_{\alpha }=\overrightarrow{\nabla }({\varphi }_{\alpha }{D}_{\alpha }\overrightarrow{\nabla }{c}_{\alpha })+P{\varphi }_{\alpha }{\varphi }_{\beta }(\frac{\partial {f}_{\beta }}{\partial {c}_{\beta }}-\frac{\partial {f}_{\alpha }}{\partial {c}_{\alpha }})+{\varphi }_{\alpha }{\dot{\varphi }}_{\alpha }({c}_{\beta }-{c}_{\alpha })$$

12 $${\varphi }_{\beta }{\dot{c}}_{\beta }=\overrightarrow{\nabla }({\varphi }_{\beta }{D}_{\beta }\overrightarrow{\nabla }{c}_{\beta })+P{\varphi }_{\alpha }{\varphi }_{\beta }(\frac{\partial {f}_{\alpha }}{\partial {c}_{\alpha }}-\frac{\partial {f}_{\beta }}{\partial {c}_{\beta }})+{\varphi }_{\beta }{\dot{\varphi }}_{\beta }({c}_{\alpha }-{c}_{\beta })$$

where *c*_*α*_ and *c*_*β*_ are the compositions of *α* and *β* phases, while *D*_*α*_ and *D*_*β*_ are the chemical diffusivities of *α* and *β* phases. The solid-liquid interface energy and interface mobility during solidification are strongly anisotropic and thus the anisotropy in the phase-field simulation of the dendritic growth needs to be taken into account. The anisotropy equation used in the phase-field simulation is as follows^13^:

13 $${\sigma }^{\ast }=\sigma (1+3{\varepsilon }_{\sigma }-4{\varepsilon }_{\sigma }({n}_{x}^{4}+{n}_{y}^{4}+{n}_{z}^{4}))$$

14 $${\mu }^{\ast }=\mu (1-3{\varepsilon }_{\mu }+4{\varepsilon }_{\mu }({n}_{x}^{4}+{n}_{y}^{4}+{n}_{z}^{4}))$$

where *σ* and *μ* are the average interface energy and interface mobility, while ${\varepsilon }_{\sigma }$ and ${\varepsilon }_{\mu }$ are the anisotropic coefficients of interface energy and interface mobility. The anisotropic coefficients of both interface energy and interface mobility were set to be 0.4. Here. ${n}_{x}$, ${n}_{y}$, ${n}_{z}$ are the normal vectors in the direction of the axis. All the material and numerical parameters used in the present three-dimensional (3-D) phase-field simulation are listed in Table 2, except for the values of the interface permeability *P*. The interfacial energy σ was directly adopted from^14^, while the interface mobility *M* was calculated according to^11^ for keeping in the diffusion-controlled regime. *Ds* and *D*_*l*_ were simply set to be constants^15^. The simulation domain was chosen to be of 128 × 128 × 128 grid points. The boundary conditions for all the phase fields and concentrations were set to be periodic.

**Table 2:** List of the numerical and materials parameters used in the present phase-field simulations.

Parameters	Symbols	Values
grid spacing	Δ*x*	50 nm
interface width	*η*	4Δ*x*
interface energy	*σ*	1.69 × 10^−5^ J/cm^2^
interface mobility	*M*	1.23 × 10^−1^ cm^4^/Js
diffusivity of liquid phase	*D*_l_	1 × 10^−5^ cm^−5^/s
diffusivity of solid phase	*D*_s_	1 × 10^−8^ cm^−5^/s

**Figure 5:**
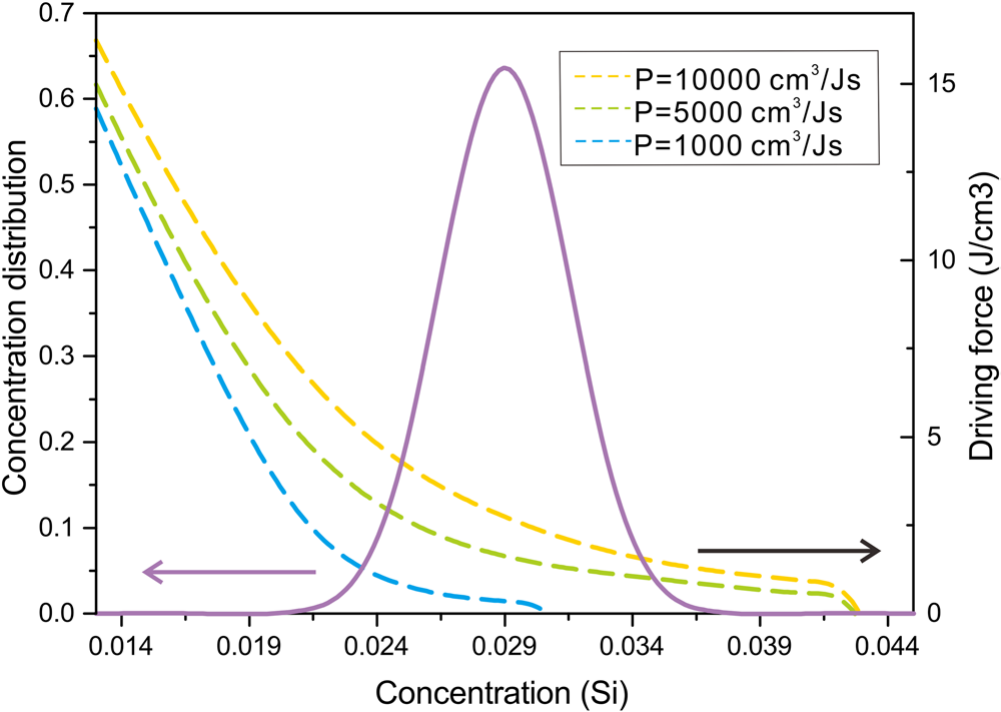
Concentration distribution in the melt and nucleation driving force with respect to different permeabilities.

First, the impact of permeability on the nucleation process is explored, as already explained above, and variation of permeability will lead to changes in the nucleus concentration and the nucleation driving force, even if the melt concentration is the same. Furthermore, the concentration distribution in the melt is represented in Fig. 5 (Note that here it is only intended to exhibit the practicability of the model, not to provide a result with high precision), and the dashed lines corresponding to different permeabilities are the calculated nucleation driving force curves. It is apparent that the nucleation driving force will be inhibited with the reduction of permeability, hence the change of permeability has noticeable impact on the nucleation events.

**Figure 6:**
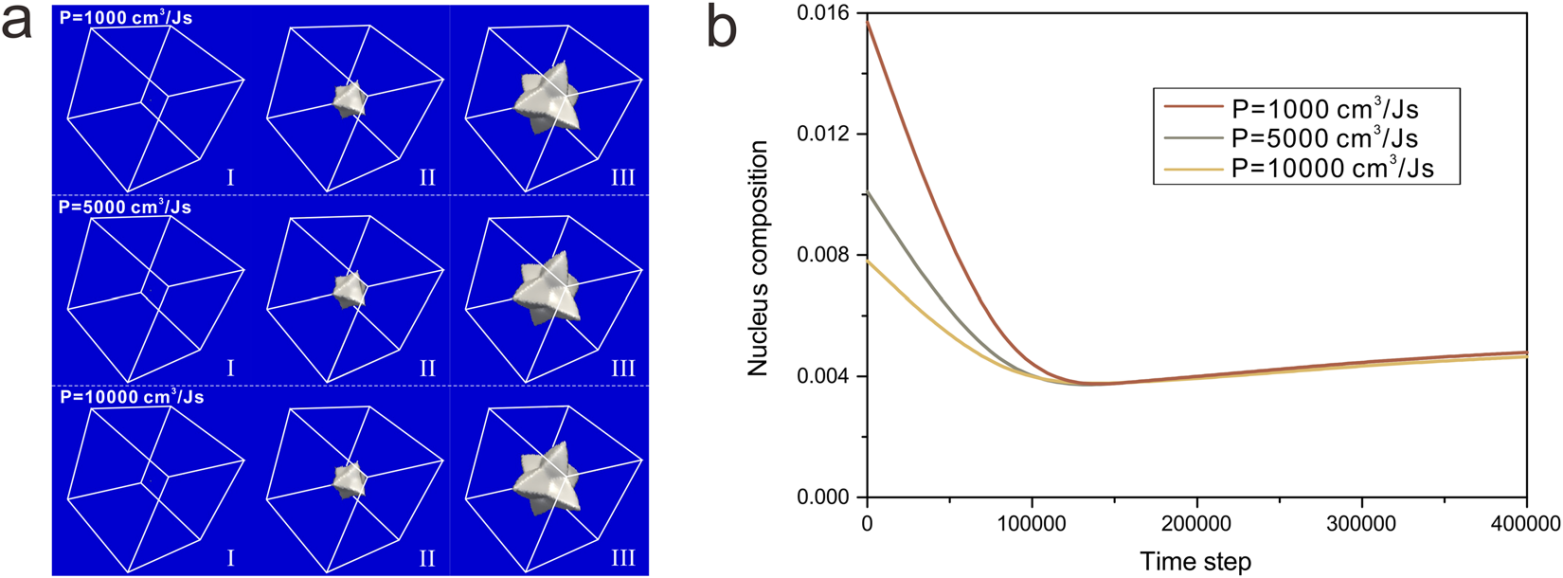
Grain growth evolution corresponding to different permeabilities during isothermal solidification (**a**) morphology evolution; (**b**) concentration evolution of the nucleus.

Then, the formation of the nucleus is followed by its dendritic growth. The 3-D phase-field simulation for grain growth evolution in Al-2.89 at. % Si alloy corresponding to different permeabilities are enumerated in Fig. 6. Figure 6(a) shows the morphology evolution of microstructure while Fig. 6(b) displays the concentration evolution of the nucleus. Obviously, the permeability has a great influence on the initial concentration of nucleus whereas it is almost impervious for both the grain growth and the subsequent concentration evolution of nucleus.

**Figure 7:**
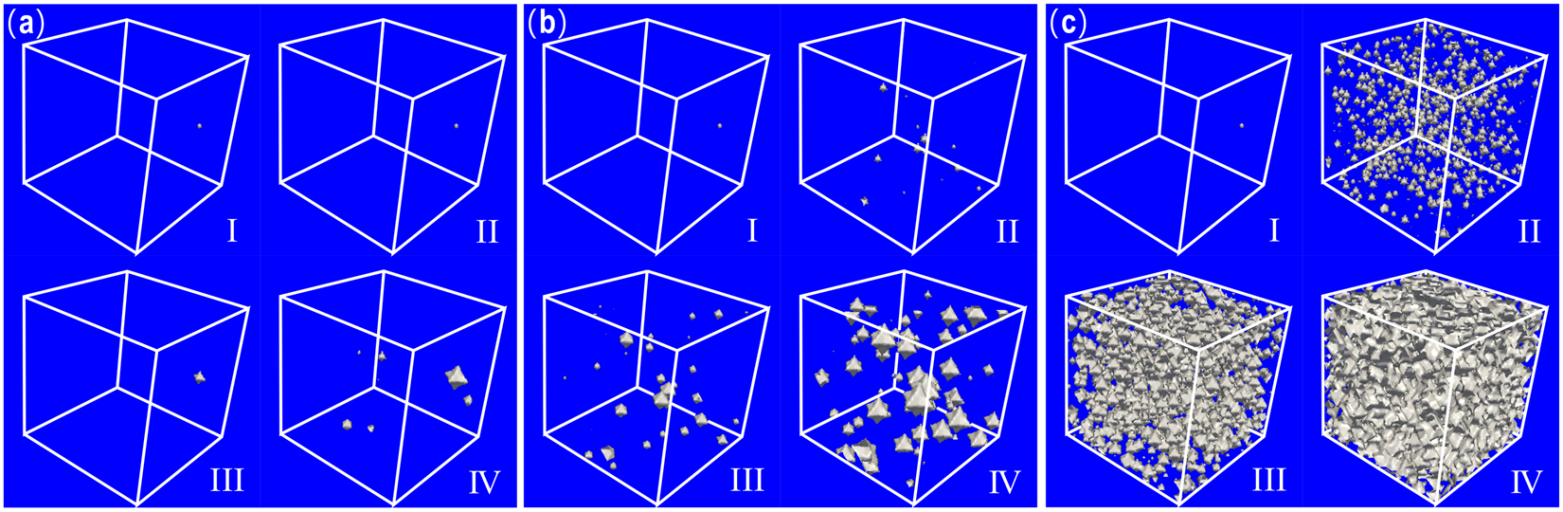
Three-dimensional phase-field simulated nucleation process of Al-2.89 at. % Si alloy during isothermal solidification with different interfacial permeabilities: (**a**) P = 1000 cm^3^/Js; (**b**) P = 5000 cm^3^/Js; (**c**) P = 10000 cm^3^/Js.

Later on, solidification processes with different interface permeabilities (i.e., 1000 cm^3^/Js, 5000 cm^3^/Js and 10000 cm^3^Js) in Al-2.89 at. % Si alloy were simulated by the 3-D phase-field model and are exhibited in Fig. 7. From the three evolutionary graphs it can be seen clearly that the nucleus are precipitated continuously with the liquid concentration undulating, nucleation is almost impossible to occur in Fig. 7(a) while the occurrence of nucleation is very easy in Fig. 7(c), thus it can be said without exaggeration that a high interface permeability is likely to increase the total number of nucleation events, fully affirmed our speculation.

Next, the nucleation rate curves corresponding to different permeabilities are fitted by a recently modified function similar to the LSW size distribution^16^:

15 $$f(n)=a\cdot {(\frac{1}{1-\tau })}^{b}\cdot {(\frac{1}{1+\tau })}^{c}\cdot \exp (-\frac{1}{1-\tau })$$

**Figure 8:**
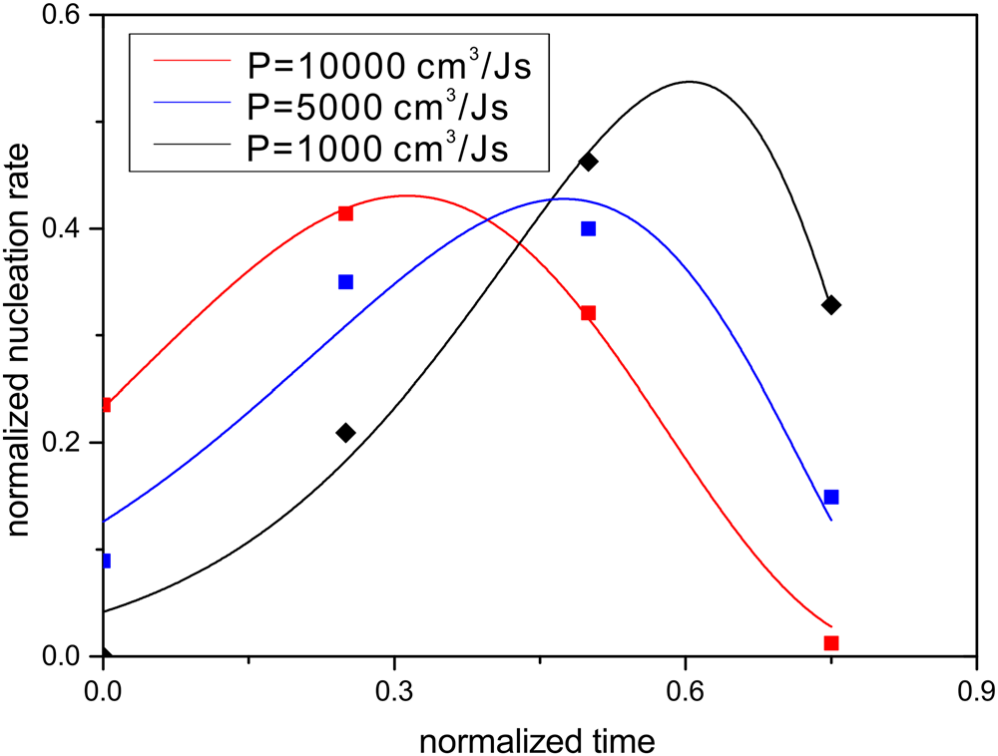
Normalized nucleation rates corresponding to different interfacial permeabilities.

Here, *a*, *b*, *c* are the adjustable parameters, the nucleation rate ($f(n)$) and the time (*τ*) are normalized values respectively (Here, the total number of nucleus are ${N}_{P=1000}^{total}=5$, ${N}_{P=5000}^{total}=67$ and ${N}_{P=10000}^{total}=1175$ respectively). As predicted in Fig. 8, the nucleation rate shows an upward trend in the beginning because a small number of nucleus will precipitate faster in the place where the driving force is larger. Then the nucleation rate shows a downward trend. The reason for this phenomenon may be that, the saturation of melt will increase with the growth of crystals, therefore the subsequent nucleation potency will be restrained.

**Figure 9:**
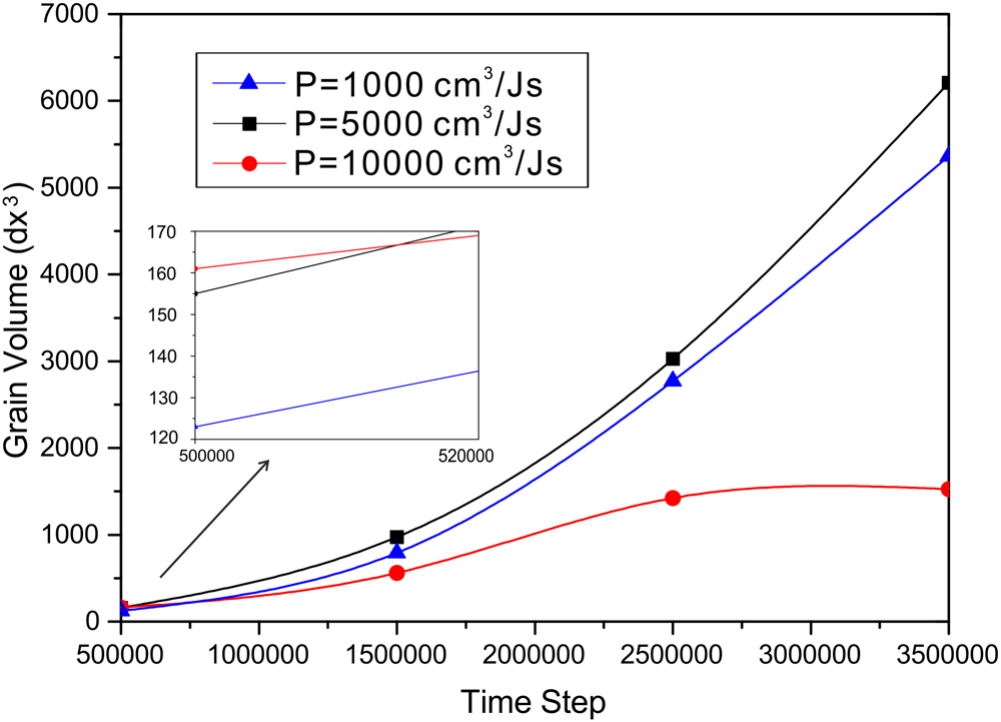
Average volume of the grains corresponding to different permeabilies.

Besides, the time to reach the peak and the quantities of nucleus are affected seriously by the permeability as well. The average volume of the grains is sketched schematically in Fig. 9, calculated by:

16 $$\bar{V}={V}_{total}/N$$

where V_total_ is the total volume of the solid phase. It can be seen clearly that at the beginning a larger permeability will result in a larger average grain volume, however, as the evolution proceeds, the average grain volume for the largest permeability is smaller compared to the other two smaller permeabilities. A feasible explanation may be that when the nucleus density is high, the growth of grains is inhibited by other grains, and with the growth of enormous amounts of nucleus, solute atoms will be released into the melt, leading to a saturation rapidly which will restrain the grain growth as well. Thus, it can be concluded that the increase in permeability can accelerate the process of solidification mainly through the promoted occurrence of nucleation.

## Conclusions

A kinetic view into the Hillert-Rettenmayr thermodynamic analysis was performed, and demonstrated to determine nucleus composition during isothermal solidification, which still represents a challenge nowadays. With the introduction of the interface permeability, the energy dissipation of the solute rearrangement at the liquid-solid interface can be evaluated, and the driving force for nucleation can be unambiguously determined.

A pragmatic nucleation model was proposed, and then validated using a 3-D phase-field simulation of nucleation and subsequent dendritic growth in one hypothetic Al-Si alloy. The simulation results indicate that the permeability affects the nucleation driving force of metastable clusters by influencing their composition, which has a great effect on the nucleation rate and finally affects the entire solidification process.
